# Case report: The spectrum of SMPD1 pathogenic variants in Hungary

**DOI:** 10.3389/fgene.2023.1158108

**Published:** 2023-06-06

**Authors:** Maria Judit Molnar, Tamas Szlepak, Ildikó Csürke, Szendile Loth, Rita Káposzta, Melinda Erdős, Antal Dezsőfi

**Affiliations:** ^1^ Institute of Genomic Medicine and Rare Disorders, Semmelweis University, Budapest, Hungary; ^2^ ELKH-SE Multiomics Neurodegeneration Research Group, Eötvös Loránd Research Network, Budapest, Hungary; ^3^Department of Pediatrics, Josa Andras County Hospital, Nyiregyhaza, Hungary; ^4^Department of Pediatrics, Semmelweis University, Budapest, Hungary; ^5^Department of Pediatrics, University of Debrecen, Debrecen, Hungary; ^6^PID Clinical Unit and Laboratory, Department of Dermatology, Venereology, and Dermatooncology, Semmelweis University, Budapest, Hungary

**Keywords:** ASMD, acid sphingomyelinase deficiency type A/B, intermediate-type acid sphingomyelinase deficiency, Niemann-Pick disease type A/B, SMPD1

## Abstract

Acid sphingomyelinase deficiency (ASMD) is an autosomal recessive disease caused by biallelic pathogenic variants in the sphingomyelin phosphodiesterase-1 (*SMPD1*) gene. Acid sphingomyelinase deficiency is characterized by a spectrum of disease and is broadly divided into three types (ASMD type A, ASMD type A/B, and ASMD type B). More than 220 disease-associated *SMPD1* variants have been reported, and genotype/phenotype correlations are limited. Here we report the first description of all six diagnosed acid sphingomyelinase deficiency cases in Hungary. Nine *SMPD1* variants are present in this cohort, including 3 *SMPD1* variants (G247D, M384R, and F572L), which have only been described in Hungarian patients. All described variants are deemed to be pathogenic. Eight of the variants are missense, and one is a frameshift variant. The treatment of an ASMD type A/B patient in this cohort using hematopoietic stem cell transplantation is also detailed. This study may help to support diagnosis, patient genetic counseling, and management of acid sphingomyelinase deficiency.

## 1 Introduction

Acid sphingomyelinase deficiency (ASMD), which was historically known as Niemann-Pick disease, is a rare, progressive, debilitating, and potentially fatal disease (McGovern et al., 2017b). ASMD is an autosomal recessive lysosomal storage disease (LSD) caused by biallelic pathogenic variants in the sphingomyelin phosphodiesterase-1 (*SMPD1*) gene, which encodes acid sphingomyelinase (ASM). Reduced levels and/or activity of ASM results in the progressive accumulation of sphingomyelin and other lipids, notably within tissues with a high reticuloendothelial cell content, such as the spleen, liver, lung, lymph nodes, and bone marrow (McGovern et al., 2017b). To date, more than 220 pathogenic sequence variants of *SMPD1* have been associated with ASMD (Hu et al., 2021). The numerous gene variants of *SMPD1*, in conjunction with other genetic and epigenetic factors, give rise to a wide range of disease symptoms and severity (McGovern et al., 2021).

ASMD is broadly divided into three types, which are characterized by their clinical manifestations, age of onset, and rapidity of disease progression (Schuchman and Desnick, 2017).

ASMD type A is an early-onset, rapidly progressing neurovisceral disorder leading to death at 2–3 years of age. These patients may exhibit symptoms including central nervous system involvement, hepatosplenomegaly, pulmonary symptoms, and a failure to thrive or reach developmental milestones (McGovern et al., 2006). ASMD type B has a slow, progressive course with limited or no neurologic involvement, and patients may survive until adolescence or adulthood. Hepatosplenomegaly is the predominant manifestation; other common symptoms include pulmonary involvement and liver dysfunction (McGovern et al., 2017a). ASMD type A/B is thought to be an intermediate clinical phenotype characterized by slow progressive, variable visceral disease and neurological degeneration (Pavlů-Pereira et al., 2005; Wasserstein et al., 2006).

Given the variety of clinical manifestations of ASMD, a wide range of clinical evaluations (including cardiac, neurological, metabolic, pulmonary, musculoskeletal, and gastrointestinal) may be undertaken before ASMD is suspected and diagnosed (McGovern et al., 2017b). The clinical diagnosis of ASMD may also be confounded by multiple symptoms that overlap with other LSDs. Consensus diagnostic guidelines for ASMD (McGovern et al., 2017b), therefore, recommend that diagnosis is made based on reduced ASM activity in whole blood, dried blood spots, fibroblasts, or leukocytes. The gold standard method of ASM estimation is tandem mass spectrometry, but fluorometric ASM activity assays are also widely used (McGovern et al., 2017b). When ASM deficiency, defined as a residual ASM less than 10% of control values (Wasserstein and Schuchman, 1993), is apparent, genetic sequencing is performed to obtain additional information and predict genotype/phenotype correlation if none has been established. Genetic sequencing may be independently diagnostic if 2 pathogenic variants are detected (McGovern et al., 2017b).

ASMD is a pan-ethnic disorder with a worldwide distribution (Simonaro et al., 2002). However, some *SMPD1* variants have a higher incidence in particular geographic regions; for example, the p.R610del variant is prevalent within the Maghreb region of North Africa (Vanier et al., 1993). Detailed studies of the ASMD genotype and phenotype have been conducted in some European areas, such as in Czech and Slovak patients (Pavlů-Pereira et al., 2005). To date, a limited amount of information on Hungarian ASMD genotypes and phenotypes is available from a research study of three Hungarian ASMD families (Tóth et al., 2011).

Here we describe the genotype and phenotype of 6 Hungarian pediatric patients and the treatment of one with a hematopoietic stem cell transplant (HSCT).

## 2 Patients and methods

Patients diagnosed with ASMD in Hungary were identified with the involvement of physicians from Hungarian Rare Disease Expert Centers and lysosomal disease treatment centers. All patients described in this case series (Table 1) are Hungarian, patients 1-5 are Caucasian, and patient 6 is of Gypsy/Roma ethnicity. *SMPD1* variants are described with reference to the GenBank (Accession Number) NM_000543.4 sequence. The impact analysis tool VarSome (Kopanos et al., 2019) and the Franklin online platform (https://franklin.genoox.com - Franklin by Genoox) were used for variant classification. ACMG (American College of Medical Genetics) classification were performed in all cases. The damaging rare variants were validated by Sanger sequencing in all cases. The trans position of the heterozygous rare variants were proven by the segregation analysis of the parents.

**TABLE 1:** Timeline, natural history, and genotype/phenotype of ASMD patients.

Patient No	1	2	3	4	5	6
Gender	Male	Male	Male	Female	Female	Male
Ethnicity	Caucasian	Caucasian	Caucasian	Caucasian	Caucasian	Roma
Disease type	ASMD Type A	ASMD Type A/B	ASMD Type A/B	ASMD Type A/B	ASMD Type B	ASMD Type B
Current age	Deceased	9 years	15 months	7 years	Deceased	Unknown
Age of disease onset	6 months	22 months	3 months	6 weeks	17 months	24 months
Presenting symptoms	Elevated liver enzymes, hepato-splenomegaly	Hepato-splenomegaly, anemia, elevated liver enzymes and thrombocytopenia	Eating difficulties, dystrophic features, axial hypotonia, slightly delayed psychomotor development, hepatosplenomegaly, and fluctuating liver enzymes	Icterus, elevated liver enzymes	Elevated liver enzymes, hepato-splenomegaly	Abdominal distension, hepato-splenomegaly
SMPD1 rare variants/ACMG classification	c.740G>A (p.G247D)/Pathogenic; c.1716C>G (p.F572L)/Likely pathogenic	c.880C>A (p.Q294K)/Pathogenic; c.518dupT (p.S174fs*19) Pathogenic	c.742G>A (p.E248K)/Pathogenic; c. 1151T>G (p.M384R)/Pathogenic	c.880C>A (p.Q294K)/Pathogenic; c.911T>C (p.L304P) Pathogenic	homozygous c.748A>C (p.S250R)/Pathogenic	homozygous c.1177T>G (p.W393G) Pathogenic
ASM activity	NA	2.5%	8%	8%	10%	NA
Age of Genetic Diagnosis	1 year	5 years	6 months	17 months	2 years	6 years
Additional Symptoms developed	Failure to thrive, severe psychomotor retardation, lymph-adenopathy, iron deficiency anemia, macrocephaly, facial dysmorphia, hypotonia, right inguinal hernia and hydrocele	Delayed motor and speech development, truncal ataxia. 4 years old, speech and mental regression. 7 years old epileptic seizures. Unable to walk due to ataxia	8 months failure to thrive, PEG tube introduced. 11 months, weight remained under the 3rd percentile, delayed motor development	2 months cholelithiasis, direct hyperbilirubinemia and hepatopathy. 4 months splenomegaly. Failure to thrive, anemia, generalized muscle hypotonia, delayed motor development, seizures, and low cortisol. 3 years pulmonary fibrosis, lung infiltration, gastroparesis, significant splenomegaly, and foamy macrophages in the bone marrow. 5 years mental regression, decreased muscle tone, delayed motor development	Nanosomia 13 years, Hypoalbuminemia, anemia, thrombo-cytopenia, and interstitial lung disease. 14 years hepatorenal syndrome, anuria, and respiratory insufficiency	Elevated liver enzymes, cherry-red spot
HSCT	N	N	N	Y at 2 years of age	N	N
Deceased	Y	N	N	N	Y	NA
Deceased age	26 month	N/A	N/A	N/A	14 years	NA

### 2.1 Case reports

All patients described in this case series (Table 1) are Hungarian, patients 1-5 are Caucasian, and patient 6 is of Romani person ethnicity. *SMPD1* variants are described with reference to the GenBank (Accession Number) NM_000543.4 sequence. The impact analysis tool VarSome (Kopanos et al., 2019) and the Franklin online platform (https://franklin.genoox.com - Franklin by Genoox) were used for variant classification (Supplementary Table S1).

Patient 1 (a male deceased at 26 months) was diagnosed at 6 months of age with ASMD type A disease, which was characterized by hepatosplenomegaly and elevated liver enzymes. He was born at term with a birth weight of 3,020 g. His perinatal adaptation was normal; no prolonged jaundice was detected. He presented with hepatosplenomegaly at the age of 6 months. Laboratory tests showed elevated liver enzymes. By 4 months, he exhibited failure to thrive and delayed motor development. From the age of 5 months, he was treated for a right inguinal hernia and hydrocele. He received iron supplementation due to iron deficient anemia. By the age of 1 year, he only learned to roll over, but could not sit, stand or walk. He had hypotonia and he could not eat chunky food. He also developed macrocephaly and facial dysmorphia characterized by distant eyes and swollen eyelids, slight exophthalmos, wide nasal roots and deep-set ears. Biallelic *SMPD1* c.740G>A (p.G247D), c.1716C>G (p.F572L) gene variants were identified at 1 year. During the second year of life, severe progression of the disease was observed, and rapid deterioration of organ functions led to death at 26 months of age.

#### 2.1.1 Patients 2–4 had ASMD A/B phenotype

Patient 2 (a 9-year-old male) was 22 months old at disease onset and presented with hepatosplenomegaly, elevated liver enzymes, anemia, and thrombocytopenia. The patient was determined (at 5 years) to have an ASMD type A/B phenotype and to carry compound heterozygous c.880C>A (p.Q294K) and c.518dupT (p.S174fs*19) *SMPD1* variants. Acid sphingomyelinase activity was decreased. Delayed motor and speech development and truncal ataxia were exhibited. At 4 years, speech and mental regression were noted, and epileptic seizures developed aged 7. The patient was unable to walk as a consequence of ataxia.

Patient 3 (a 15-month-old male) presented at 3 months with eating difficulties, dystrophic features, axial hypotonia, slightly delayed psychomotor development, hepatosplenomegaly, and elevated liver enzymes. Ferritin was 502 μg/L. C-triol (cholestane-3β,5α,6β-triol) was 109 ng/ml (normal range 0–40 ng/mL), 7-ketocholesterol: 395 ng/ml (normal range 0–85 ng/mL). Acid sphingomyelinase activity was decreased at 0.1 μmol/L/h (cut off > 1.2). He was determined (at 6 months) to have compound heterozygous c.742G>A (p.E248K) and c.1151T>G (p.M384R) *SMPD1* variants. By 8 months, he exhibited a failure to thrive, and a PEG tube was introduced. At 11 months, his weight remained under the 3rd percentile, and he has delayed motor development. He is able to roll over in both directions but cannot crawl. He does not exhibit any pulmonary symptoms.

Patient 4 (a 7-year-old female) first exhibited symptoms of icterus and elevated liver enzymes at 6 weeks. She demonstrated cholelithiasis, direct hyperbilirubinemia and hepatopathy (at 2 months), and splenomegaly (at 4 months). Liver enzymes were elevated. Subsequently, failure to thrive, anemia, generalized muscle hypotonia, delayed motor development, seizures, and low cortisol were noted. Her acid sphingomyelinase activity was decreased at 0.1 μmol/L/h (cut off > 1.2). Genetic analysis (at 17 months of age) revealed biallelic c.880C>A (p.Q294K) and c.911T>C (p.L304P) *SMPD1* variants. At 2 years of age, the patient received an allogeneic bone marrow transplant. However, donor chimerism rapidly decreased in the peripheral blood. Approximately 6 weeks after the initial procedure, an additional “mega-dose” haploidentical transplant with continual G-CSF was required. Three months later, a (CD 20 negative, mucosa-associated lymphoid tissue-type) post-transplant lymphoproliferative disorder developed, which was successfully treated with dexamethasone. A bone marrow biopsy confirmed normal thrombopoiesis; however, a low peripheral platelet count was noted, which may have been a consequence of hypersplenism. At 3 years of age, signs of disease progression were observed, including pulmonary fibrosis, lung infiltration, gastroparesis, significant splenomegaly, and foamy macrophages in the bone marrow. At 5 years, she is still PEG tube fed and demonstrates some mental regression, decreased muscle tone, and delayed motor development.

#### 2.1.2 Patient 5–6 were classified as ASMD type B

Patient 5 (a female deceased at 14 years of age) was born at term with a birth weight of 2,750 g. Her perinatal adaptation was normal, no prolonged jaundice was detected. She presented with elevated liver enzymes and hepatosplenomegaly at 17 months. She also had nanosomia. Laboratory tests showed elevated liver enzymes. She had significantly decreased acid sphingomyelinase activity (0.24 μkat/kg protein, normal range: 2.3–3.9 μkat/kg protein). Genetic analysis (conducted at 2 years) revealed this patient was homozygous for the c.748A>C (p.S250R) *SMPD1* variant. She had no neurological disease manifestations. Hypoalbuminemia, anemia, thrombocytopenia, and interstitial lung disease were noted at 13 years, and hepatorenal syndrome, anuria, and respiratory insufficiency developed by 14 years of age. The patient died aged 14 years due to severe hepatic dysfunction, hepatorenal syndrome, interstitial lung disease, and cardiac failure.

Patient 6 (male) displayed abdominal distension and hepatosplenomegaly at 24 months. He had no neurological disease manifestations at diagnosis. Liver enzymes were elevated, and a cherry-red spot was noted. This patient is Roma origin and is homozygous for the c.1177T>G (p.W393G) *SMPD1* variant. A genetic diagnosis was established at 6 years of age. Limited further clinical information is available for this patient.

## 3 Discussion

Here we describe the genotype and phenotype of 6 Hungarian ASMD patients. Nine *SMPD1* variants are present in this cohort. To date, 3 *SMPD1* variants identified in this study (G247D, M384R, and F572L) have solely been described in Hungarian patients. The remaining *SMPD1* variants have been previously documented in Bulgarian, Jewish, Chinese, Korean, Italian, Czech, and Slovakian patients (Levran et al., 1992; Ferlinz et al., 1995; Simonaro et al., 2002; Harzer et al., 2003; Sikora et al., 2003; Ricci et al., 2004; Pavlů-Pereira et al., 2005; Mihaylova et al., 2007; Cho et al., 2009; Gan-Or et al., 2013; Zampieri et al., 2016; Kaseniit et al., 2020; Hu et al., 2021). Eight of the variants are missense, which are deemed to be pathogenic, and one is a frameshift variant (Zampieri et al., 2016). Several of the missense variants present in this cohort of patients have been functionally evaluated (G247D, Q294K, L304P, W393G, and F572L), which demonstrated a range of residual activity from less than 5% up to 30% of wild-type ASM activity (Tóth et al., 2011; Zampieri et al., 2016). Frameshift variants are deleterious as they can produce mRNA, which is eliminated by nonsense-mediated decay or lead to the generation of truncated and potentially afunctional proteins (Zampieri et al., 2016). Frameshift variants generally result in limited or no ASM activity (McGovern et al., 2017b). However, the frameshift variant (p.S174fs*19) identified in this case series has not been further characterized.

Genetic testing revealed that the only patient diagnosed with ASMD type A (patient 1) carried p.G247D and p.F572L *SMPD1* variants. This patient was previously reported by Tóth et al. (2011). Their functional *in vitro* analysis demonstrated that both variants (but notably p.G247D) decreased the half-life and catalytic activity of ASM, which may explain the severe and ultimately fatal ASMD disease trajectory in p.G247D, p.F572L patients (Tóth et al., 2011).

One of the ASMD type B patients described here (patient 6) is of Roma origin and homozygous for the p.W393G variant. A study of 20 Bulgarians with Roma ancestry showed that all the individuals were homozygous for the p.W393G variant and had an ASMD A/B phenotype. These patients demonstrated a wide spectrum of neurological involvement (Mihaylova et al., 2007). Functional studies of the resultant protein demonstrated limited ASM stability which may underpin the limited sphingomyelinase activity in these patients (Ferlinz et al., 1995). These data suggest that this variant is a Roma founder mutation.

Two patients in this case series carry the p.Q294K variant in heteroallelic form either alongside the p.L304P variant [ASMD type A/B (patient 4)]; or the p.S174fs*19 frameshift variant in the intermediate ASMD type A/B participant (patient 2). The p.L302P (also known as p.L304P) variant accounted for ∼24% of the mutant alleles in a group of Ashkenazi Jewish ancestry who had ASMD type A (Levran et al., 1992). Q294K is a common *SMPD1* gene variant that is strongly associated (in either homoallelic or heteroallelic form) with neurological symptoms and disease progression (Pavlů-Pereira et al., 2005; Wasserstein et al., 2006). When p.Q294K is present in heteroallelic form, the phenotype is modified by the second *SMPD1* variant (Pavlů-Pereira et al., 2005). A study in Czech and Slovak patients revealed that p.Q294K is one of the most common variants in Eastern Europe (Pavlů-Pereira et al., 2005). The p.Q294K variant was previously identified in the Hungarian population by anonymous retrospective testing of newborn screening cards for LSDs (Wittmann et al., 2012). However, this study was not designed to provide information on phenotype and disease trajectory or allow for patient follow-up (Wittmann et al., 2012).

An ASMD type A/B patient in this case series (patient 3) is heteroallelic for the p.E248K missense variant (Ricci et al., 2004), which is thought to be a functionally important variant as it resulted in charge changes (Ricci et al., 2004). p.E248K has been previously documented in Korean (Cho et al., 2009), Italian (Ricci et al., 2004), and Chinese (Hu et al., 2021) patients.

The Hungarian ASMD type B patient homozygous for p.S250R (patient 5) died aged 14. The p.S250R variant was also present in heteroallelic form (alongside p.Q294K) in a previously described 4-year-old patient who exhibited massive hepatosplenomegaly, lung infiltration but no neurological symptoms (Harzer et al., 2003). However, no follow-up information on patient longevity is provided (Harzer et al., 2003).

The most frequently reported worldwide *SMPD1* variant is c.1829_1831del (p. R610del) (Zampieri et al., 2016). Interestingly it was not present in any of the Hungarian patients studied. However, Eastern European populations may have a lower incidence of the p.R610del variant, given that it was not identified in a cohort of patients from the Czech Republic (Pavlů-Pereira et al., 2005; Zampieri et al., 2016).

Until early 2022, when enzyme replacement therapy for ASMD was initially approved (Keam, 2022), no disease-specific treatments for ASMD were available. Limited reports document the successful use of bone marrow or stem cell transplantation in ASMD (Victor et al., 2003; Shah et al., 2005). Despite the use of 2 allogeneic stem cell transplants in patient 4, the (p.Q294K; p.L304P) ASMD type A/B patient described, disease progression has continued.

In conclusion, this case report represents the most comprehensive description of ASMD in Hungary to date. Nine *SMPD1* variants are present in this cohort (Supplementary Table S1), including 3 *SMPD1* variants (p.G247D, p.M384R, and p.F572L), which have solely been described in Hungarian patients. In patients who present with rare or novel *SMPD1* variants, genotyping has limited prognostic value. Therefore, the analysis of possible correlations between the genotype and ASMD disease course can inform clinical management and help to develop novel or personalized therapies.

## Data Availability

The datasets for this article are not publicly available due to concerns regarding participant/patient anonymity. Requests to access the datasets should be directed to the corresponding author.
