# Supplementary material for: Case report: The spectrum of SMPD1 pathogenic variants in Hungary
Source: Front Genet. 2023 Jun 6;14:1158108. doi: 10.3389/fgene.2023.1158108 (PMC10280011; doi:10.3389/fgene.2023.1158108)
Supplement: Supplementary file 1 [file Table1.docx]

Supplemetary Table 1.

**The list of rare variants and their frequency in gnomAD and ACMG classification**

| **Variant** | **Clinvar ID** | **gnomAD freq (v2.1.1)** | **In silico prediction** | | **ACMG classification** |
| --- | --- | --- | --- | --- | --- |
|  |  |  | Franklin | VarSome |  |
| p.S174fs*19 | 189153 | 0.000004044 | No prediction^1^ | No prediction^1^ | Franklin Pat/Varsome Pat |
| p.G247D | 813476 | Not in the database | Deleterious | Strong pathogenic | Franklin LikelyP/Varsome Pat |
| p.E248K | 496823 | 0.000003570 | Deleterious | Moderate pathogenic | Franklin Pat/Varsome Pat |
| p.S250R | 371576 | 0.000008030 | Uncertain | Uncertain significance | Franklin Pat/Varsome Pat |
| p.Q294K | 2994 | 0.000007955 | Deleterious | Moderate pathogenic | Franklin Pat/Varsome Pat |
| p.L304P | 2989 | 0.00001591 | Deleterious | Moderate pathogenic | Franklin Pat/Varsome Pat |
| p.M384R | Not in the database | Not in the database | Deleterious | Strong pathogenic | Franklin LikelyP/ Varsome Pat |
| p.W393G | 2991 | 0.000004010 | Deleterious | Strong pathogenic | Franklin Pat/Varsome Pat |
| p.F572L | Not in the database | Not in the database | Deleterious | Moderate pathogenic | Franklin VUS/Varsome LikelyP |

^1^ Null variants in a gene with known LoF mechanism of a disease apply ACMG rule PVS1 that disables rule PP3, therefore it does not calculate in silico prediction score for that variant.
